# Frequency of symptoms, determinants of severe symptoms, validity of and cut-off score for Menopause Rating Scale (MRS) as a screening tool: A cross-sectional survey among midlife Nepalese women

**DOI:** 10.1186/1472-6874-11-30

**Published:** 2011-06-14

**Authors:** Neena Chuni, Chandrashekhar T Sreeramareddy

**Affiliations:** 1 Department of Obstetrics and Gynaecology, Manipal Teaching Hospital, Manipal College of Medical Sciences, Pokhara, Nepal; 2 Department of Obstetrics and Gynaecology, Indira Gandhi Memorial Hospital, Male, Maldives; 3 Department of Community Medicine, Manipal Teaching Hospital, Manipal College of Medical Sciences, Pokhara, Nepal; 4 Department of Community Medicine, Melaka Manipal Medical College, Jalan Batu Hampar, Bukit Baru, Melaka, Malaysia

## Abstract

**Background:**

Majority of Nepalese women live in remote rural areas, where health services are not easily accessible. We determined the validity of Menopause Rating Scale (MRS) as a screening tool for identification of women with severe menopausal symptoms and cut-off MRS score for referral.

**Methods:**

A cross-sectional survey was carried out between February and August, 2008. Trained health workers administered MRS and a questionnaire to 729 women (40 to 65 years) attending health screening camps in Kaski district of Western Development Region of Nepal. Information about demographics, menopausal status, and use of hormone replacement therapy (HRT), chronic disease, self-perceived general health and reproductive history was also collected. Menopausal status was classified according to the Staging of Reproductive Ageing Workshop (STRAW). We calculated rates of menopausal symptoms, sensitivity, and specificity and likelihood ratios of MRS scores for referral to a gynaecologist. We also carried out multivariate analyses to identify the predictors for referral to a gynaecologist for severe symptoms.

**Results:**

A total 729 women were interviewed. Mean age at menopause was 49.9 years (SD 5.6). Most frequently reported symptoms were, sleeping problems (574, 78.7%), physical and mental exhaustion (73.5%), hot flushes (508, 69.7%), joint and muscular discomfort (500, 68.6%) and dryness of vagina (449, 61.6%). Postmenopausal women (247, 33.9%) and perimenopausal (215, 29.5%) women together experienced significantly higher prevalence of all symptoms than the premenopausal (267, 36.6%) women. MRS score of ≥16 had highest ratio for (sensitivity + specificity)/2. Women who reported urogenital symptoms [OR 5.29, 95% CI 2.59, 10.78], and self perceived general health as poor [OR 1.29, 95% CI 1.11, 1.53] were more likely to be referred to a gynaecologist for severe menopausal symptoms. While women reporting somatic [OR 0.72, 95% CI 0.63, 0.82] and psychological [OR 0.86, 95% CI 0.74, 0.99] symptoms were less likely to be referred.

**Conclusion:**

MRS may be used as a screening tool at a cut-off score of ≥16 with least misclassification rate. However, its utility may be limited by woman's general health status and occurrence of urogenital symptoms.

## Background

Menopause is a condition caused by the depletion of ovarian function followed by cessation of menstruation in women. Modern medicine has significantly prolonged the life span of humans and most women spend one-third to half of their lifetime in post- menopause [1]. Information about menopausal experiences among different racial and ethnic groups is important for healthcare personnel to provide appropriate and specific interventions [2,3]. It has been shown that menopausal symptoms vary according to racial groups. For instance, studies have reported that somatic and psychological symptoms are less frequent among Asian women as compared to Caucasian women [4-7]. Further, menopausal symptoms may also vary according to menopausal status. Vasomotor, sexual and psychological symptoms are more frequent among perimenopausal and postmenopausal women [8-10].

During menopause, women often experience some symptoms which may affect their daily activities. In recent years, studies have shown that menopausal symptoms may affect health-related quality of life [11,12]. Menopause Rating Scale (MRS) which is a health related quality of life (HRQOL) scale was developed in the early 1990's in Germany [13,14]. Since then, MRS has been well accepted internationally and has been translated into several languages [15] taking international methodological recommendations into consideration [16]. Use of MRS in Turkish language has been validated [17] and also used as an instrument to assess the frequency menopausal symptoms among middle aged women in eastern Malaysia, Northern India, Sri Lanka and Ecuador [8,10,18,19]. MRS has a potential of being used as a screening tool to identify those women in need of referral to higher level for severe menopausal symptoms. Such use of MRS would be more appropriate in remote and rural areas of Nepal. In rural Nepal, health care facilities are less accessible to women due to geographic, cultural and social barriers. In such settings, the community health workers may administer MRS to identify the women who are in need of secondary or tertiary level care for severe menopausal symptoms. Therefore it is important to test the validity of MRS as a screening tool. We aimed to determine the frequency of menopausal symptoms among rural Nepalese women; to test the validity of MRS as a screening tool for identification of severe menopausal symptoms; and to determine the cut-off MRS score for referral to specialist consultation. To the best of our knowledge, such study has not been carried out in Nepal.

## Methods

### Study design

A cross-sectional, interviewer administrated questionnaire survey

### Setting and participants

Kaski district is one of the 14 districts in Western Development Region (WDR) of Nepal. In WDR of Nepal most districts are rural and remote, where access to healthcare services and information is very limited. Kaski district has a land area of 2000 squares kilometres and a population of 380, 000. Kaski district has 43 villages and Pokhara sub metropolitan city which has a population of 156, 000 according to the 2001 census [20]. In Nepal, healthcare is offered mainly through Primary Health Centres (PHCs), operated by the Ministry of Health and manned by General Practitioners (GPs), Auxiliary Nurse Midwives (ANMs), Auxiliary Health Workers (AHWs) and health assistants (HAs). Bedabari PHC is adjacent to Pokhara city and Batulechaur Health Post (HP) is a under Bedabari PHC. Each PHC serves a population of 100,000 and HP serves 30,000 population. This study was carried out at afore-mentioned health facilities and the participants were women aged between 40 and 65 years who voluntarily attended the health screening camps.

### Instrument

The questionnaire was divided into four sections (additional files 1 and 2). The first section included the following information about women's socio-demographic characteristics: age, marital status, living situation, education and occupation. The second section included questions about menopausal status at the time of the study, menstrual history and symptoms of dysmenorrhoea experienced in the past. Menopausal status was defined according to STRAW (Stages of Reproductive Aging Workshop) classification. STRAW categorises and defines menopausal women as follows: Premenopause: minor changes in cycle length particularly decreasing length of the cycle. Late perimenopause: had menstruation during the past 2-12 months but not during the past two months. Early perimenopause: had increasing irregularity of menses without skipping periods (7 days difference from the beginning of a given cycle to the next) experienced after the previously regular cycle. Postmenopausal: no menstrual bleeding during the past 12 months [21,22]. Early and late perimenopause were combined into perimenopausal stage for our analysis. The third section included questions about presence of any chronic diseases (diabetes, hypertension, bronchial asthma, cardiac disease etc), use of HRT and the women were asked to rate self-perceived general health and wellbeing as good or poor. In the last section women were asked if they had experienced any symptoms based on the MRS, since the age of 40 years. We used the English version of MRS without modification. The original German version of MRS has been translated into English and other languages. In all versions, the MRS is a self-administered questionnaire and is widely tested and accepted internationally [13-16]. During the interview women were asked to report symptoms and also asked to rate the severity of symptoms as 'mild', 'moderate', 'severe' or 'very severe' [14]. If any of the symptoms listed in MRS were not reported by women then it was marked as 'none'. MRS score was generated by summing the score given for each of the 11 symptoms. Scoring for each symptom was given as follows: none = 0, mild = 1, moderate = 2, severe = 3, very severe = 4.

### Ethics and informed consent

This survey was approved by the research ethics committee of Manipal College of Medical Sciences, Pokhara, Nepal. The women were provided with information about nature of interview to be conducted and participation was voluntary. They were assured about confidentiality of the information to be provided. Informed consent was taken from each eligible woman before the questionnaire interview was administered.

### Data collection

During February 2008 to August 2008, health screening camps were held in Bedabari PHC and Batulechaur HP. We included all women aged between 40 to 65 years. The women were checked for the exclusion criteria during registration process at the reception desk of the camps. The exclusion criteria were the following: pregnant and lactating mothers, women with history of cancer in remission or under treatment currently; history of alcohol or drug abuse and any mental disability or undergoing treatment for psychiatric disorders. Women with premature ovarian failure or known genital malformations were also excluded. If the woman was found eligible, she was invited to participate in the study. To each eligible woman explanation about purpose of the interview was given and informed consent was taken. A face-to-face interview was conducted by paramedical health personnel who were trained about the questionnaire and the English version of MRS. All the interviews took place in the local language, Nepalese.

### Statistical Analysis

Data was coded and entered into Microsoft Excel. The data was converted into SPSS and STATA packages for analysis. We used descriptive statistics to summarise the demographic variables. Frequency of occurrence and severity (as severe and very severe) of symptoms were calculated as percentages. We compared mean ages, MRS scores and severity of symptoms between premenopausal, perimenopausal and postmenopausal status of the women according to STRAW classification. Total MRS Score, and sub scale scores for somatic, psychological and urogenital symptoms were calculated separately. We used non-parametric receiver operating characteristic (ROC) curves command in STATA (version10) to calculate the sensitivity, specificity, positive predictive values and likelihood ratios for different MRS scores to identify the women who are likely be referred to a gynaecologist for severe menopausal symptoms. By this method, we also calculated area under curve (AUC) and its 95% confidence intervals (95% CIs) for total MRS score. We also carried out a multivariate analysis on SPSS package (version 14) to find out the predictors for referral to a gynaecologist. Referral to a gynaecologist at least once was considered as a dependant variable. Age, self-reported general health, presence of any chronic disease, regularity of menstrual cycles, history of dysmenorrhoea, history of abortions and scores of MRS sub scales i.e. scores for somatic, psychological and urogenital symptoms were treated as dependant variables.

## Results

### Response rates and demographic characteristics

During the survey period, 1179 women attended the health screening camps. When the eligibility criteria were applied 18 women were either pregnant or were lactating, one woman had received treatment for a genital malignancy and one woman had reached premature menopause (i.e. before 40 years of age). Ninety four women had undergone treatment for chronic psychiatric illness of whom, 62 were chronic alcoholics. Thus 1065 eligible women were invited to participate in the survey interview but 336 women declined to participate. The main reasons for refusal were the need to return back home quickly or non-comprehension about the nature of interview to be carried out. We interviewed 729 women giving a response rate of 68.5% (729/1065). Demographic characteristics of the women are shown in table 1. Mean age of the women interviewed was 49.9 years (SD = 5.6). Median age was 49 years (lower quartile i.e. Q1 & upper quartile i.e. Q3 were 46 and 53 years respectively). Mean age of the women according to menopausal status (classified according to STRAW) were as follows: premenopausal 45.1 years (SD = 2.78), perimenopausal 49.14 years (SD = 2.01), postmenopausal 55.67 years (SD = 5.6). Majority (88.6%) of the women were currently married. Of these, 574 (78.7%) women were living with their husbands. The women we interviewed were mostly illiterates (468, 64.9%) and mainly housewives (528, 72.4%).

**Table 1:** Socio-demographic profile of the participants

Characteristic	Number	Percentage
**Age**		

40-44	130	17.8

45-49	269	36.9

50-54	195	26.7

55-59	70	9.6

60-64	65	8.9

**Marital status**		

Currently married	646	88.6

Divorced	28	3.8

Widowed	54	7.4

**Education level**		

Illiterate	468	64.9

Primary level	121	16.6

Secondary level	93	12.8

Tertiary level	47	6.4

**Living situation**		

With partner	574	78.7

With children/others	150	20.6

Alone	4	0.5

**Occupation **		

Housewife	528	72.4

General worker	154	21.1

Semi professional	42	5.8

Professional	5	0.7

### Health status and frequency of menopausal symptoms

One hundred and eighty six women (25.5%) rated their general health as poor, while 127 (17.4%) women had one or more chronic disease. The main chronic diseases the women had were Chronic Obstructive Pulmonary Disease (58), hypertension (34), diabetes (26), others (9) Seventy five (10.3%) women reported that they had visited a gynaecologist for menopausal symptoms and 21 (2.9%) of them had undergone hormone replacement therapy. Frequency and severity of the menopausal symptoms are shown in table 2. Most frequent somatic symptoms were sleeping problems, (574, 78.7%) hot flushes (508, 69.7%) and joint and muscular discomfort (500, 68.6%). Among psychological symptoms, physical and mental exhaustion were reported by 536 (73.5%) women. Among the urogenital symptoms, dryness of vagina was reported by 449 (61.6%) women and 385 (52.8%) women reported bladder problems. Among all the menopausal symptoms, hot flushes were perceived as severe (157, 21.5%) and very severe (75, 10.3%). Other symptoms that were perceived as severe were: anxiety (114, 15.6%), depressive mood (109, 14.9%), irritability (105, 14.4%), dryness of vagina (102, 13.9%). The differences between three groups of menopausal status for all the symptoms were statistically significant (Table 3). MRS score including the sub scale scores and severity of symptoms were significantly higher among postmenopausal women than the perimenopausal and premenopausal women (table 4).

**Table 2:** Frequency and severity of menopausal symptoms

Menopausal symptoms	Number (%)	Severe	Very severe
**Somatic**			

Sleeping problems	574 (78.7)	93	1

Hot flushes, sweating	508 (69.7)	157	75

Joint and muscular discomfort	500 (68.6)	86	0

Heart discomfort	360 (49.4)	0	0

**Psychological**			

Physical and mental exhaustion	536 (73.5)	68	4

Depressive mood	402 (55.2)	109	12

Irritability	339 (46.5)	105	10

Anxiety	334 (45.8)	114	7

**Urogenital**			

Dryness of vagina	449 (61.6)	102	4

Bladder problems	385 (52.8)	38	2

Sexual problems	343 (47.1)	56	6

**Table 3:** Distribution of menopausal symptoms according to menopausal status

Menopausal symptoms	All (%) N = 729	Premenopause N = 267	Perimenopause N = 215	Post menopause N = 247
**Somatic**				

Sleeping problems	574 (78.7)	160 (59.9)	185 (86.1)	229 (92.7)

Hot flushes, sweating	508 (69.7)	61 (22.8)	204 (94.9)	243 (98.4)

Joint and muscular discomfort	500 (68.6)	130 (48.7)	167 (77.7)	203 (82.2)

Heart discomfort	360 (49.4)	81 (30.3)	111 (51.6)	168 (68.0)

**Psychological**				

Physical & mental exhaustion	536 (73.5)	153 (57.3)	171 (79.5)	212 (85.8)

Depressive mood	402 (55.2)	112 (41.9)	138 (64.2)	152 (61.5)

Irritability	339 (46.5)	60 (22.5)	134 (62.3)	145 (58.7)

Anxiety	334 (45.8)	90 (33.7)	104 (48.4)	140 (56.7)

**Urogenital**				

Dryness of vagina	449 (61.6)	61 (22.8)	173 (80.5)	215 (87.1)

Bladder problems	385 (52.8)	92 (34.5)	128 (59.5)	165 (66.8)

Sexual problems	343 (47.1)	111 (41.6)	166 (77.2)	199 (80.6)

* All comparisons were statistically significant (p < 0.01) by chi square test

**Table 4:** Comparison of MRS scores and severe menopausal symptoms according to the menopausal status

Variable	Premenopause N = 267	Perimenopause N = 215	Postmenopause N = 247	p-value
Total MRS score (Mean & SD)	5.3 (3.79)	12.28 (3.36)	16.24 (4.81)	< 0.001*

Somatic symptoms (Mean & SD)	2.17 (2.06)	5.27 (1.96)	7.17 (2.34)	< 0.001*

Psychological symptoms (Mean & SD)	1.82 (1.31)	3.61 (1.60)	4.36 (2.12)	< 0.001*

Urogenital symptoms (Mean & SD)	1.30 (1.51)	3.39 (1.70)	4.72 (2.11)	< 0.001

Severe symptoms (Number and percentage)	8 (2.9)	41 (19.1)	154 (62.3)	< 0.001 α

* ANOVA test α Chi square test

### Predictors of severe menopausal symptoms

Table 5 shows the predictors for referral to a gynaecologist for evaluation of severe menopausal symptoms by univariate and multivariate analyses. On univariate analysis, self-reported poor general health, presence of chronic disease, irregular menstrual cycles, dysmenorrhoea and occurrence of all three groups of menopausal symptoms were associated with referral to a gynaecologist. On multivariate analysis, women reporting poor general health (OR 5.29, 95% CI 2.59 10.78) were likely to be referred. Among menopausal symptoms women reporting somatic (OR 0.72, 95% CI 0.63 0.82) and psychological (OR 0.86, 95% CI 0.74 0.99) symptoms were less likely to be referred. Women reporting urogenital (OR 1.29, 95% 1.11 1.53) symptoms were more likely to be referred to a gynaecologist for further evaluation.

**Table 5:** Multivariate analysis of predictors of referral to a gynaecologist for evaluation of severe menopausal symptoms

Variable	Univariate OR (95% CI)	p-value	Adjusted OR (95% CI)	p-value
**Age**	0.89 (0.86, 0.93)	< 0.001	1.01 (0.94, 1.07)	0.859

**General health**				

Good	1		1	

Poor	10.16 (5.93, 17.43)	< 0.001	**5.29 (2.59, 10.78)**	**< 0.001**

**Chronic disease**				

No	1		1	

Yes	1.86 (1.07, 3.23)	0.028	1.54 (0.78, 3.04)	0.21

**Regular menstrual cycles**				

Yes	1		1	

No	6.63 (2.83, 15.48)	< 0.001	0.53 (0.19, 1.46)	0.217

**Dysmenorrhea**				

No	**1**		**1**	

Yes	2.04 (1.05, 3.96)	0.035	0.97 (0.44, 2.15)	0.939

**Abortions**				

None	1		1	

One or more	1.25 (0.74, 2.10)	0.402	0.90 (0.49, 1.64)	0.736

**Menopausal symptoms**				

Somatic symptoms	0.64 (0.57, 0.71)	< 0.001	**0.72 (0.63, 0.82)**	**< 0.001**

Psychological symptoms	0.67 (0.60, 0.76)	< 0.001	**0.86 (0.74, 0.99)**	**0.046**

Urogenital symptoms	0.81 (0.73, 0.89)	< 0.001	**1.29 (1.11, 1.53)**	**0.001**

### Validity of MRS as a screening tool

**Figure 1:**
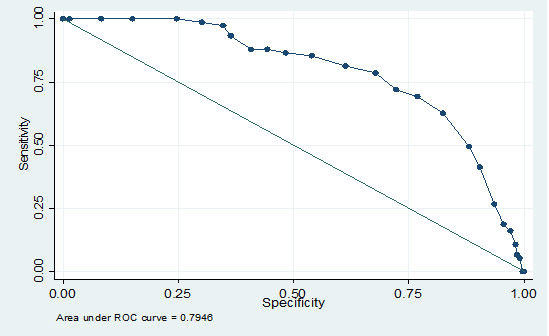
**Receiver operator characteristic curve to determine the cut-off point to MRS score for referral to a gynaecologist**.

The sensitivity, specificity, positive predictive values and likelihood ratios for different MRS scores to identify the women who are likely to be referred to a gynaecologist for severe menopausal symptoms are shown in table 6 and in Figure 1 by non-parametric receiver operating characteristic (ROC) curves. The area under the curve (AUC) was calculated using the non-parametric method of De Long. We chose an optimum cut-off point at a total MRS score where the ratio (sensitivity + specificity)/2 was highest. At this point the lowest total misclassification error rate was expected [23]. This criterion was chosen to allow for comparison with previous studies available in the literature [24]. The optimal cut-off MRS score for referral to gynaecologist was 16. The area under curve was 0.79 (95% CI 0.74 0.84).

**Table 6:** Sensitivity, Specificity, positive predictive values and likelihood ratios for predicting the women who should likely be referred to a gynaecologist for evaluation of severe menopausal symptoms

Cut-off point	Sensitivity	Specificity	Correctly classified	LR+	LR-
( > = 1 )	100.00%	0.00%	10.29%	1	

( > = 2 )	100.00%	1.38%	11.52%	1.014	0

( > = 3 )	100.00%	8.26%	17.70%	1.09	0

( > = 4 )	100.00%	14.98%	23.73%	1.1763	0

( > = 5 )	100.00%	24.62%	32.37%	1.3266	0

( > = 6 )	98.67%	30.12%	37.17%	1.412	0.0443

( > = 7 )	97.33%	34.71%	41.15%	1.4908	0.0768

( > = 8 )	93.33%	36.39%	42.25%	1.4673	0.1832

( > = 9 )	88.00%	40.83%	45.68%	1.4871	0.2939

( > = 10 )	88.00%	44.34%	48.83%	1.5811	0.2706

( > = 11 )	86.67%	48.32%	52.26%	1.6769	0.2759

( > = 12 )	85.33%	53.98%	57.20%	1.8541	0.2717

( > = 13 )	81.33%	61.31%	63.37%	2.1025	0.3044

( > = 14 )	78.67%	67.74%	68.86%	2.4383	0.3149

( > = 15 )	72.00%	72.32%	72.29%	2.6015	0.3871

( > = 16 )	69.33%	76.91%	76.13%	3.0029	0.3987

( > = 17 )	62.67%	82.42%	80.38%	3.5638	0.453

( > = 18 )	49.33%	88.07%	84.09%	4.1364	0.5753

( > = 19 )	41.33%	90.37%	85.32%	4.2908	0.6492

( > = 20 )	26.67%	93.58%	86.69%	4.1524	0.7837

( > = 21 )	18.67%	95.57%	87.65%	4.2097	0.8511

( > = 22 )	16.00%	97.09%	88.75%	5.5074	0.8651

( > = 23 )	10.67%	98.17%	89.16%	5.8133	0.91

( > = 24 )	6.67%	98.47%	89.03%	4.36	0.9478

( > = 25 )	5.33%	99.08%	89.44%	5.8134	0.9554

( > = 26 )	0.00%	99.69%	89.44%	0	1.0031

( > = 28 )	0.00%	99.85%	89.57%	0	1.0015

( > 28 )	0.00%	100.00%	89.71%		1

## Discussion

Our health facility-based survey among midlife women with an interviewer administered MRS could identify the frequency and severity of menopausal symptoms among rural and largely illiterate women. MRS fared well as a screening instrument and a total MRS score of 16 and above could be considered as an optimal cut-off point for referral to a gynecologist. However, sub scale score for urogenital symptoms was a better predictor for referral to a gynaecologist. The optimistic results of our survey on a relatively large sample of women should be interpreted carefully in the light of some limitations we had. The sample of women we surveyed, were from catchment area of two health facilities where the women's health camps were held. This makes our sample non-representative of Nepalese women, thus limiting the external validity. Further, all women may not have correctly recalled the symptoms listed in the MRS and about referral to a gynaecologist for evaluation of severe menopausal symptoms. There may also have been an element of subjectivity while assessing the severity of symptoms. Response rates were not high mainly due to crowding caused during free of cost health camps and lack of comprehension by lesser educated women. So there may have been some selection bias.

The prevalence and proportion of menopausal symptoms may vary according to racial and ethnic groups. Population-based surveys among Caucasian populations have reported a higher prevalence (40 -70%) [25-27] while those from Asian countries have reported a lower prevalence (10-50%) [6]. Three studies from Turkey have reported varying prevalence (35-90%) [28-30]. In our survey, though all symptoms were reported, the proportion of women reporting symptoms listed in MRS varied. Sleep disorders, physical and mental exhaustion were most common symptoms. Prevalence of hot flushes and sweating in our survey was nearly same as prevalence reported from western countries. Though hot flushes was not a common symptom was perceived as 'severe' and 'very severe'. Hot flushes, sweating, vaginal dryness and sleep disturbances are considered the main climacteric complaints in western countries [31-33]. Similarly, hot flushes and sweating were the most common complaints among Turkish women [34]. In our survey, hot flushes and sleep disorders were the most common symptoms among both perimenopausal and postmenopausal women. These symptoms can be explained by the physiological fluctuations in oestrogen levels. Similar results were reported from studies carried out among Caucasian, Australian and South-east Asian women [35,39]. The probability for occurrence of other symptoms is higher if the woman had experienced vasomotor symptoms [40]. Several studies from Asian countries have also reported a lower prevalence of classical menopausal symptoms [35-38,41-44]. Such variability of menopausal symptoms is not well known. This may be due to woman's attitude and awareness, or socio-cultural and economic factors.

Symptoms reported during mid-life may also be influenced by various factors [45]. These may be due to increased levels of physical and mental stress requiring support and coping mechanisms. Socio-cultural milieu and economic factors may also affect these support and coping mechanisms. Reports suggest that menopausal symptoms may also be confounded by symptoms attributable to genetic factors and aging per se [32]. Therefore, the symptoms reported may not be attributable to climacteric itself. This argument may be supported by our results, where the commonest symptoms reported by premenopausal women were physical and mental exhaustion and sleep disorders. All premenopausal women had reported symptoms given in the MRS questionnaire. Our findings support the view that symptoms experienced by these women may have originated from physical and psychological problems the women had but not as a menopausal symptom alone [1,46]. The proportion of women reporting their general health as poor and visiting a gynaecologist for their menopausal symptoms was only a tenth. However the same proportions were nearly a third in studies reported from Turkey [30,47].

On multivariate analysis, women who reported their general health as poor were likely to be referred to a gynaecologist for evaluation. Similar results have been reported by other studies [17,18,48]. We found that women reporting urogenital symptoms were more likely to be referred to a gynaecologist for further evaluation. Women with somatic and psychological symptoms were less likely to be referred to a gynaecologist for evaluation of symptoms. Women with psychological and somatic symptoms may not have attributed their symptoms to menopause. As we discussed above these somatic and psychological symptoms may be as result of physical and psychological stress, these rural women experience during midlife. Therefore, the women may have over-rated their symptoms as menopausal symptoms when MRS was administered to them. On the contrary to the report from Turkey, presence of a chronic disease was not a predictor for referral to a gynaecologist. We expect that self-reported health status and presence of chronic disease may have confounded the predictors of referral for severe menopausal symptoms. Use of MRS instrument as a screening tool may be affected by these confounding factors as discussed above. Urogenital symptoms seem to be more specific than the psychological and somatic symptoms. This result should be verified by future studies minimising the misreporting of menopausal symptoms when MRS is administered. The optimal cut-off for MRS score we obtained for referral to a gynaecologist was ≥16 and the sensitivity and specificity values at this cut-off are comparable to those reported from MRS validation study [49] and indicate a better reliability than a similar study from reported from Turkey [17]. The method we adopted to determine the cut-off score was in accordance to literature available about validation of screening instruments in rural areas. To our knowledge, there are very few studies which have tested the validity of MRS as screening tool. The potential utility of MRS as a screening instrument to be administered by paramedical workers should be further established. However, such potential utility appears to be limited by misclassification of somatic and psychological symptoms arising from other causes as 'menopausal'. Utility of MRS as a screening instrument should be carefully considered to avoid too many false positives being referred for evaluation of severe menopausal symptoms. Such excess referrals may cause an additional burden on medical personnel in resource-limited rural settings like Nepal.

## Conclusion

The symptoms reported by the women according to MRS were concordant to the existing literature about the utility of MRS to assess menopausal symptoms. Though symptoms reported were similar to those reported by women from other Asian countries, there may have been over-reporting of some psychological and somatic symptoms. Urogenital symptoms appear to be more specific for referral to a gynaecologist. Further studies are required to confirm or refute the utility of MRS as a screening instrument.

## Competing interests

The authors declare that they have no competing interests.

## Authors' contributions

NC conceived the study, designed the questionnaire, collected the data and co-drafted the manuscript for publication. CTS contributed to the study design, conducted data analysis and interpreted the results and co-drafted manuscript for publication. Both authors read and approved the final manuscript to be submitted for publication.

## Pre-publication history

The pre-publication history for this paper can be accessed here:

http://www.biomedcentral.com/1472-6874/11/30/prepub

## Supplementary Material

Additional file 1 **Questionnaire used for the survey Socio-demographic information, Reproductive history, STRAW classification and chronic disease information**. Click here for file

Additional file 2 **English Version of Menopause Rating Scale**. Eleven questions about menopausal symptoms and their severity in a five point Likert scale. Click here for file
