# Supplementary material for: Frequency of symptoms, determinants of severe symptoms, validity of and cut-off score for Menopause Rating Scale (MRS) as a screening tool: A cross-sectional survey among midlife Nepalese women
Source: BMC Womens Health. 2011 Jun 14;11:30. doi: 10.1186/1472-6874-11-30 (PMC3126771; doi:10.1186/1472-6874-11-30)
Supplement: Additional file 1 — Questionnaire used for the survey Socio-demographic information, Reproductive history, STRAW classification and chronic disease information. [file 1472-6874-11-30-S1.DOC]

**Assessment of Menopausal Symptoms using Menopause Rating Scale (MRS)**

**Serial number:__________**

**Demographic data**

Age in completed years:_________

Marital status: Married_____ Divorced_______ Widowed_______

Living situation: With partner_____ With children/others______ alone ________

Education level: Illiterate____ Primary level­­­­_______ Secondary level____ Tertiary level_______

Occupation: Housewife_____ General Worker____ Semi professional________Professional_____

**Reproductive history**

Age at menarche_____years, Age at 1^st^ delivery___years, Age most recent delivery_____years

Number of Pregnancies________ Number of Deliveries______ Number of Miscarriages_______

**Menstrual/menopausal history**

During last 12 months did you have menstrual cycles? Yes____ No_____

If yes, are your cycles coming regularly? Yes____ No_____

Classification of menopausal status according to STRAW: Please tick the appropriate category

Premenopausal_____ Perimenopausal____ Postmenopausal___

Did you ever have dysmenorrhoea? Yes____ No_____

Did you ever use Hormone Replacement Therapy? Used in the past______ Never used______

Have you gone to a gynaecologist’s check up for menopause symptoms in the past?

At least once_____ Never_____

How do you rate your health in general? Good____________ Poor________________

Are you currently suffering from any of the following diseases?

Hypertension_____Diabetes____ Bronchial Asthma_______ Any other_______________

Proceed to next page for assessment of menopausal symptoms using menopause rating scale.
