# Supplementary material for: Frequency of symptoms, determinants of severe symptoms, validity of and cut-off score for Menopause Rating Scale (MRS) as a screening tool: A cross-sectional survey among midlife Nepalese women
Source: BMC Womens Health. 2011 Jun 14;11:30. doi: 10.1186/1472-6874-11-30 (PMC3126771; doi:10.1186/1472-6874-11-30)
Supplement: Additional file 2 — English Version of Menopause Rating Scale. Eleven questions about menopausal symptoms and their severity in a five point Likert scale. [file 1472-6874-11-30-S2.PDF]

Menopause Rating Scale (MRS)

Which of the following symptoms apply to you at this time? Please, mark the appropriate box for each symptom. For symptoms that do not apply, please mark 'none'.

**Symptoms:**	**none**	**mild**	**moderate**	**severe**	**very severe**
**Score =**	**0**	**1**	**2**	**3**	**4**
1. Hot flushes, sweating (episodes of sweating)	☐	☐	☐	☐	☐
2. Heart discomfort (unusual awareness of heart beat, heart skipping, heart racing, tightness)	☐	☐	☐	☐	☐
3. Sleep problems (difficulty in falling asleep, difficulty in sleeping through, waking up early)	☐	☐	☐	☐	☐
4. Depressive mood (feeling down, sad, on the verge of tears, lack of drive, mood swings)	☐	☐	☐	☐	☐
5. Irritability (feeling nervous, inner tension, feeling aggressive)	☐	☐	☐	☐	☐
6. Anxiety (inner restlessness, feeling panicky)	☐	☐	☐	☐	☐
7. Physical and mental exhaustion (general decrease in performance, impaired memory, decrease in concentration, forgetfulness)	☐	☐	☐	☐	☐
8. Sexual problems (change in sexual desire, in sexual activity and satisfaction)	☐	☐	☐	☐	☐
9. Bladder problems (difficulty in urinating, increased need to urinate, bladder incontinence)	☐	☐	☐	☐	☐
10. Dryness of vagina (sensation of dryness or burning in the vagina, difficulty with sexual intercourse)	☐	☐	☐	☐	☐
11. Joint and muscular discomfort (pain in the joints, rheumatoid complaints)	☐	☐	☐	☐	☐
